# Association of perivascular fat density with vulnerable plaque and recent symptomatic cerebrovascular events

**DOI:** 10.3389/fcvm.2026.1965601

**Published:** 2026-10-20

**Authors:** Jun-Yan Yue, Hai-Peng Liu, Ran Li, Kai Ji, Zhan Yin, Song-Ge Ding, Chang-Hua Liang

**Affiliations:** 1 Department of Radiology, the First Affiliated Hospital of Henan Medical University, Xinxiang, China; 2 Department of Radiology, General Hospital of Pingmei Shenma Group, Pingdingshan, China; 3 MRI Department, the Nanyang Central Hospital, Nanyang, China

**Keywords:** black-blood image, carotid atherosclerosis, perivascular fat density, tomography, vulnerable plaque

## Abstract

**Objective:**

The aim of this study was to investigate the association between perivascular fat density (PFD) and carotid plaque characteristics and to evaluate the discriminative ability of PFD and lipid-rich necrotic core plaques in relation to symptomatic status of cerebrovascular events.

**Methods:**

A retrospective cross-sectional analysis was conducted in 119 patients with unilateral carotid atherosclerosis. Patients were classified into symptomatic and asymptomatic groups based on the occurrence of acute ischemic stroke or transient ischemic attack within the preceding two weeks. Differences in plaque characteristics, PFD, lipid-rich core plaques, and geometric parameters between the two groups were compared using non-parametric statistical tests. Spearman rank correlation analysis was used to evaluate the association between PFD and plaque characteristics. Multivariable logistic regression analysis was performed to identify risk factors and develop discriminative models. Internal validation was performed using bootstrap-derived optimism-corrected AUC.

**Results:**

Statistically significant differences in plaque characteristics, PFD, lipid-rich necrotic core plaques, and geometric parameters were observed between the symptomatic and asymptomatic groups (*p* < 0.001). PFD was positively correlated with plaque characteristics (*p* < 0.001). PFD, degree of stenosis, and the presence of lipid-rich core plaques were identified as independent correlates of cerebrovascular events. Receiver operating characteristic curve analysis demonstrated that the combined model improved the area under the curve from 0.885 for the stenosis-only model to 0.972. Calibration analysis demonstrated good agreement between model-estimated probabilities and observed outcomes, and decision curve analysis indicated greater net clinical benefit for the combined model than for the stenosis-only model.

**Conclusion:**

PFD is positively associated with carotid plaque characteristics and may serve as an imaging marker for assessing the perivascular inflammatory state. Combining PFD with stenosis severity and LRNC plaques demonstrated high discriminative value in assessing the symptomatic status of recent cerebrovascular events.

## Introduction

Stroke is the second leading cause of death worldwide, and ischemic stroke is the most common subtype, accounting for approximately 69.6%–72.8% of all stroke cases (1, 2). Ischemic stroke has a complex and multifactorial etiology, with cardio embolism, large artery atherosclerosis, small vessel occlusion, and cryptogenic stroke representing its principal causes (3–5). Among these etiologies, carotid atherosclerosis accounts for approximately 20%–30% of ischemic strokes and represents a major cause of cerebrovascular events (6, 7). Compared with other etiologies, carotid atherosclerotic plaques are distinguished by well-defined features of plaque vulnerability, making them particularly prone to erosion, rupture, and subsequent distal embolization (8, 9). Among these, thin-cap fibroatheroma, characterized by a large lipid-rich necrotic core (LRNC), a thin fibrous cap (thickness < 65 μm), and active inflammation, is considered the most unstable and rupture-prone lesion. Plaque rupture may release embolic material into the cerebral circulation, resulting in acute ischemic cerebrovascular events (10). Unlike cardio embolism, which requires anticoagulation therapy, or small vessel disease, which primarily requires intensive vascular risk factor control, the identification of vulnerable carotid plaques directly influences decisions regarding antiplatelet therapy, lipid-lowering treatment, and the timing of carotid revascularization (11, 12). Accurate characterization of plaque vulnerability at an early stage remains essential for stroke risk stratification, the development of secondary prevention strategies, and individualized treatment planning.

High-risk plaque features, including LRNC, a thin or ruptured fibrous cap, and intraplaque hemorrhage, are significantly associated with subsequent ischemic cerebrovascular events (13). High-resolution magnetic resonance vessel wall imaging (HRMR-VWI) is considered an important technique for the assessment of plaque composition; however, its widespread clinical application is limited by relatively long acquisition times, broad range of contraindications, and relatively high cost, particularly in primary care hospitals, large-scale population-based screening, and emergency evaluation (14). Compared to HRMR-VWI, computed tomography angiography (CTA) has become an important imaging modality for the clinical evaluation of carotid artery disease because of its wide availability, rapid acquisition, and relatively few contraindications (15). In the acute stroke care pathway, CTA can be performed immediately after non-contrast CT has excluded intracranial hemorrhage (16). However, its ability to identify non-calcified vulnerable plaque components remains limited because of partial-volume effects and overlapping attenuation values among plaque constituents (17). Improving the capability of CTA to identify vulnerable plaque features while maintaining its established clinical advantages has become an important area of investigation.

Recent advances in computed tomography post-processing have led to the development of black-blood computed tomography (BBCT), which combines flexible image registration with subtraction techniques and delayed contrast enhancement imaging (18). Because the extracellular space within fibrous tissue and the plaque matrix is relatively large, contrast material is retained for a longer duration, resulting in characteristic delayed enhancement. Through flexible registration and subtraction of arterial-phase and delayed-phase images, BBCT eliminates interference from high-density intraluminal contrast material while preserving delayed enhancement within the vessel wall and plaque tissues (18, 19). BBCT has demonstrated a sensitivity of 90% and a specificity of 93% for identifying vulnerable plaques, supporting its utility for plaque characterization (20).

Interleukin-6 (IL-6) and C-reactive protein (CRP) are well-established biomarkers for inflammation and are used to assess inflammatory activity and disease prognosis (21, 22). During the progression of vulnerable carotid plaques, serum levels of IL-6 and CRP increase, making them useful biomarkers for evaluating inflammation, disease severity, and treatment response (23, 24). However, as IL-6 and CRP are influenced by a variety of inflammatory and non-inflammatory conditions, they lack specificity for the assessment of plaque inflammation (25, 26). Consequently, non-invasive imaging biomarkers capable of more specifically reflecting local vascular inflammation are needed. Perivascular adipose tissue (PVAT), the specialized adipose tissue surrounding blood vessels, has emerged as a potential imaging target for this purpose (27). On computed tomography, the characteristics of PVAT can be quantified as perivascular fat density (PFD) (28). Through paracrine signaling, PFD can indirectly reflect the inflammatory status of the vessel wall, providing a non-invasive imaging biomarker of vascular inflammation associated with vulnerable plaques (29). PFD has been reported to be significantly and positively correlated with CRP and IL-6 and to be independently associated with internal carotid artery stenosis and vulnerable plaque characteristics, suggesting that PFD may provide an important link between imaging findings and systemic inflammatory status (30). To date, research on PFD has focused predominantly on coronary artery disease (CAD), whereas studies involving the carotid arteries remain relatively limited in both number and methodological quality (27, 31).

The aim of this study was to investigate the association between PFD and vulnerable carotid plaque characteristics identified using BBCT and to evaluate the incremental discriminative value of combining PFD, LRNC and stenosis severity in identifying patients with recent symptomatic cerebrovascular events.

## Materials and methods

### Study population

This retrospective cross-sectional study included consecutive patients with suspected carotid atherosclerosis who underwent imaging evaluation at the First Affiliated Hospital of Henan Medical University between December 2023 and June 2025.The inclusion criteria were as follows: (1) age 18–85 years, (2) completion of triphasic CTA of the carotid bifurcation, (3) availability of BBCT images for reconstruction, (4) unilateral carotid atherosclerosis，and (5). The exclusion criteria were as follows: (1) ipsilateral intracranial artery stenosis >50%, (2) previous carotid artery stenting or endarterectomy, (3) near-occlusion or occlusion of the internal carotid artery, (4) non-atherosclerotic vascular diseases (e.g., intracranial aneurysm, moyamoya disease, Takayasu arteritis, or intracerebral hemorrhage), (5) poor image quality that precluded evaluation, (6) cerebrovascular events with a definite embolic source or attributable to other non-atherosclerotic causes, and (7) cerebrovascular events not located in the territory supplied by the carotid artery lesion. The study protocol was approved by the Ethics Committee of the First Affiliated Hospital of Henan Medical University (approval No. EC-024-215), and the requirement for informed consent was waived owing to the retrospective study design. Acute ischemic stroke was defined as a neurological deficit persisting for more than 24 h or as acute cerebral infarction confirmed by magnetic resonance imaging. Transient ischemic attack (TIA) was defined as transient focal neurological dysfunction lasting <24 h without evidence of permanent tissue injury and manifesting as transient vision loss, unilateral sensory abnormalities, or mild hemiparesis (32). The diagnosis of stroke or TIA was established by a senior neurologist based on clinical symptoms, time of symptom onset, and imaging findings. Patients were classified into symptomatic and asymptomatic groups according to the presence or absence of ipsilateral acute ischemic stroke or TIA within the two weeks preceding CTA examination. Clinical variables, including age, sex, body mass index, hypertension, diabetes mellitus, CAD, hyperlipidemia, and smoking history, were collected.

### CTA protocol

Triphasic head and neck CTA was performed using a 320-detector row CT scanner (Aquilion ONE, Canon Medical Systems). Patients were positioned supine and instructed to avoid swallowing and to keep their head still during image acquisition. Non-contrast and arterial-phase images were acquired from the aortic arch to the skull base. The scanning parameters were as follows: tube voltage, 100 kV; automatic tube current modulation; rotation time, 0.35 s; and pitch, 1.388. Following acquisition of the non-contrast CT images, an iodinated contrast agent (iopromide, 370 mg/mL; Jiangsu Hengrui Pharmaceuticals Co., Ltd.) was administered through the right median cubital vein using a dual-head power injector at a flow rate of 5 mL/s, with a total volume of 80–100 mL (1.5 mL/kg), followed by a 20 mL saline flush at the same injection rate. Delayed-phase imaging of the carotid bifurcation was performed 90 s after contrast administration. This delay time was selected according to the routine delayed-phase imaging protocol for carotid artery evaluation, which is widely used in clinical practice for optimal visualization of the vessel wall and plaque components. All CT images were reconstructed using a hybrid iterative reconstruction algorithm, with a standard soft-tissue kernel. BBCT images were subsequently generated using dedicated artificial intelligence (AI)-assisted flexible registration software (CE-boost, SURESubtraction Iodine Map), which applies differential flexible subtraction to suppress intraluminal contrast enhancement, following the standardized automated workflow integrated within the software.

### Image analysis

BBCT and conventional CTA images were analyzed using the PACS system (V2.35S, Shenzhen Zuobiao Software). All images were independently evaluated by two radiologists (with 2 and >5 years of radiology experience, respectively), both of whom were blinded to the patients’ clinical symptoms. In cases of disagreement, the final decision was made by a third radiologist with more than 10 years of experience. Representative measurements are illustrated in Figure 1.

**Figure 1:**
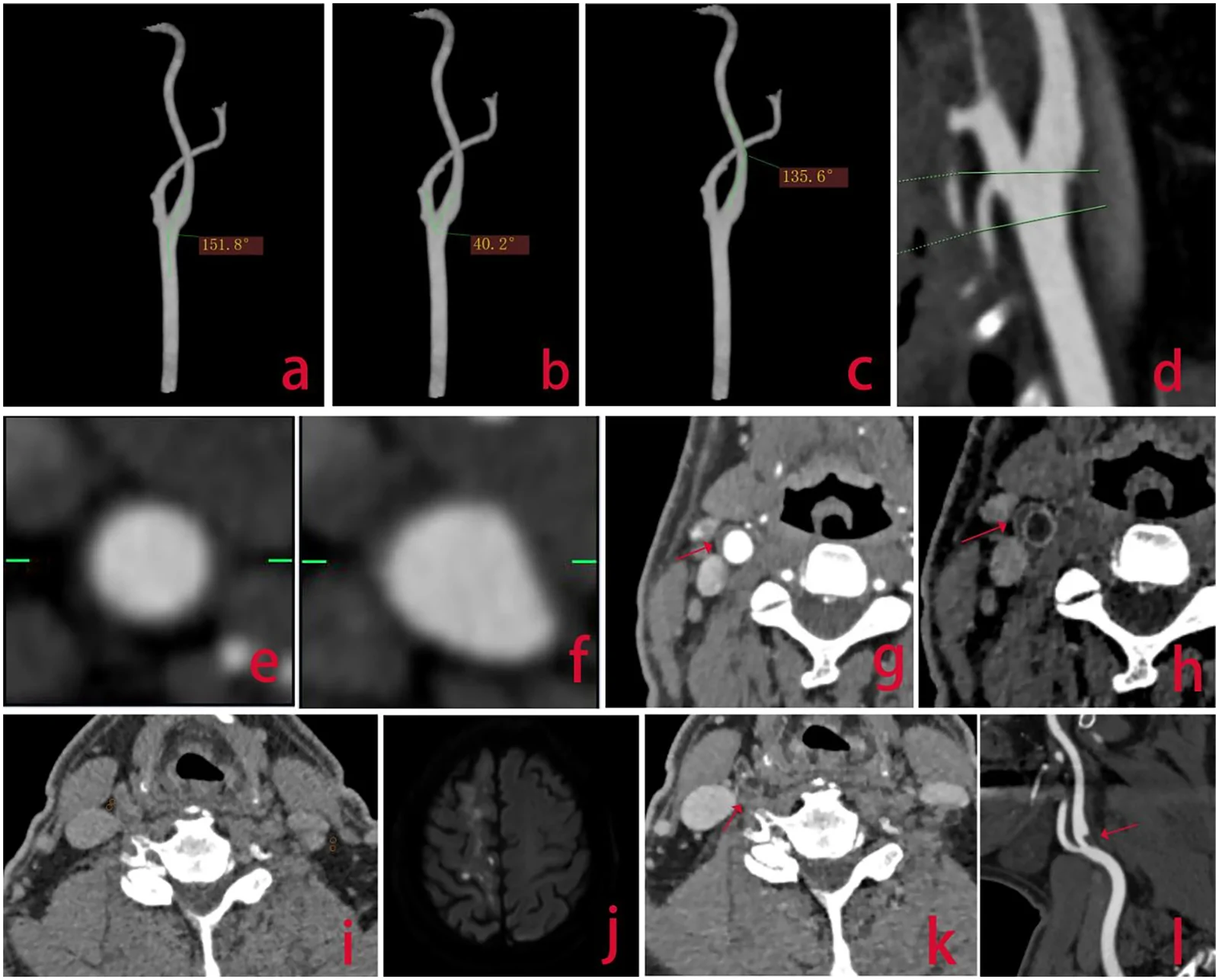
Representative measurements and imaging findings. **(a)** CCA–ICA angle. **(b)** Bifurcation angle. **(c)** Proximal ICA curvature angle. **(d–f)** Cross-sectional vessel area measurements at the CCA bifurcation. **(g,h)** Lipid-rich necrotic core plaques. **(i)** ROI delineation for measurement of PFD. **(j–l)** Representative images from a 68-year-old man presenting with dizziness and limb weakness.

### Plaque characteristics

The degree of stenosis was assessed according to the North American Symptomatic Carotid Endarterectomy Trial criteria (33, 34).

Stenosis (%) = (1—diameter at the narrowest point/diameter of the normal carotid artery distal to the stenotic lesion) × 100%.

Maximum plaque thickness was defined as the greatest plaque thickness measured on axial CTA images (35). The eccentricity index was calculated as 1—(minimum wall thickness/maximum wall thickness). Plaque burden was calculated as (1—lumen area/vessel area) × 100%. The remodeling index was defined as (vessel area at the site of maximal stenosis/reference vessel area at the distal segment) × 100%.

### Geometric parameters

The common carotid artery-internal carotid artery (CCA–ICA) angle was measured on longitudinal reformatted images as the angle between the centerlines of the proximal ICA and the distal CCA. The proximal ICA curvature angle was measured at the first bend of the cervical ICA (C1 segment) as the angle formed by lines connecting the ICA centerline on either side of the curvature. The carotid bifurcation angle was defined as the angle between the ICA and ECA centerlines. The CCAmax/CCA ratio was calculated as the ratio of the maximum vessel area at the carotid bifurcation to the vessel area at the origin of the CCA.

### LRNC and PFD

LRNC plaque was defined as a plaque in which the LRNC occupied >40% of the total plaque area on BBCT images (10), and the use of BBCT for plaque component characterization has been validated in our previous work (36). PFD measurements were performed on non-contrast CT images. On axial images, ROIs were manually placed within the perivascular adipose tissue surrounding the carotid plaque, at least 1 mm beyond the outer vessel wall boundary, with care taken to avoid the lumen and adjacent bony structures. Measurements were repeated three times, and the average value was used for analysis, consistent with previously reported methodologies. Two elliptical ROIs were manually delineated and adjusted according to the plaque location and the distribution of the surrounding adipose tissue. The maximum and mean CT attenuation values were recorded in Hounsfield units, and the average values obtained from the two ROIs were used for analysis. To ensure consistency in ROI size, shape, and location on the contralateral side, the same-slice sequential method was applied using the PACS system.

### Statistical analysis

Statistical analyses were performed using IBM SPSS Statistics version 26.0 (IBM Corp., Armonk, NY, USA). No missing data were present for any of the variables in this study. The Kolmogorov–Smirnov test and Bartlett's test were used to assess the normality of data distribution and the homogeneity of variances. Continuous variables were expressed as mean ± standard deviation (x¯ ± s), and categorical variables were expressed as frequencies (percentages). Non-parametric tests were used to compare plaque characteristics, PFD, LRNC, and geometric parameters between the symptomatic and asymptomatic groups. The intraclass correlation coefficient (ICC) was used to evaluate intra-observer and inter-observer agreement, with an ICC >0.75 indicating good reliability. For categorical variables such as plaque type, inter-observer agreement was assessed using the Kappa statistic, with a Kappa value > 0.80 considered to indicate excellent agreement. Spearman rank correlation analysis was performed to evaluate the correlation between PFD and plaque characteristics. All non-baseline study variables were entered into a multivariable logistic regression model to identify independent correlates of cerebrovascular events. Multicollinearity was assessed using correlation coefficients and variance inflation factors (VIFs).

Four models were constructed based on the degree of stenosis, LRNC, and PFD: stenosis alone, stenosis plus LRNC, stenosis plus PFD, and a combined model including stenosis, LRNC, and PFD. Model performance was evaluated using receiver operating characteristic (ROC) curve analysis and the corresponding area under the curve (AUC). Internal validation was performed using bootstrap-derived optimism-corrected AUC, with 1000 bootstrap resamples. Model calibration was assessed using the Hosmer–Lemeshow goodness-of-fit test and clinical utility was evaluated using decision curve analysis (DCA).

## Results

Reproducibility of the imaging measurements was assessed prior to the main analyses. For the primary continuous PFD parameters, the intra-observer and inter-observer ICCs were 0.937 (95% CI: 0.910–0.955) and 0.859 (95% CI: 0.803–0.899), respectively, for PFDmax, and 0.899 (95% CI: 0.858–0.928) and 0.842 (95% CI: 0.781–0.887), respectively, for PFDmean, indicating good to excellent reliability. For categorical variables (e.g., LRNC), the inter-observer Kappa value was 0.834 (*p* < 0.05), indicating excellent agreement.

A total of 886 patients were screened, of whom 119 met the eligibility criteria and were ultimately included in the analysis (Figure 2). Among these, 67 (56.3%) were assigned to the asymptomatic group and 52 (43.7%) to the symptomatic group. Baseline demographic and clinical characteristics are presented in Table 1.

**Figure 2:**
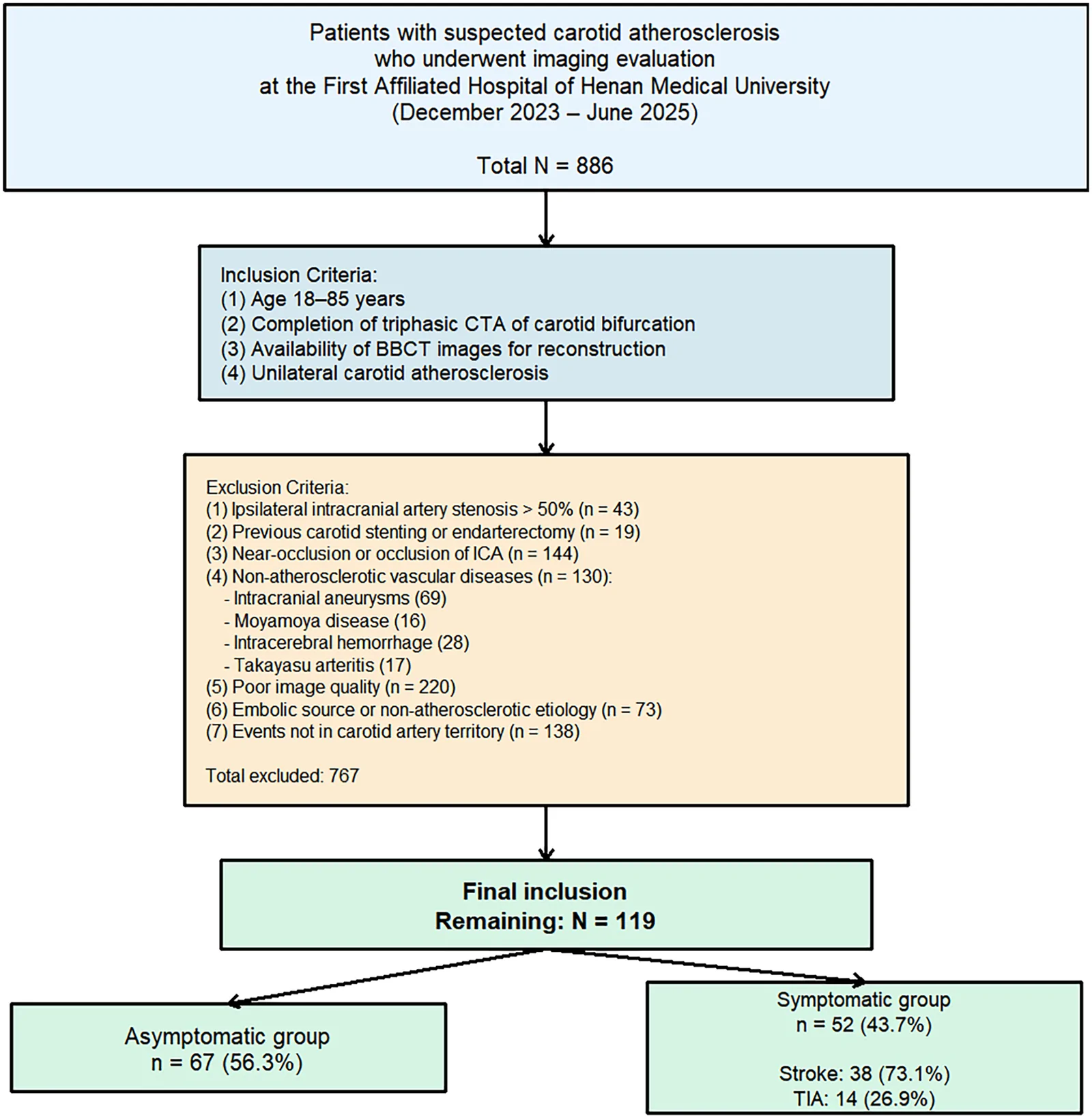
Study inelusion and exclusion flow chart.

**Table 1:** Comparison of baseline data between the symptomatic group and the asymptomatic group.

Variables	Total(*n* = 119)	Symptomatic(*n* = 52)	Asymptomatic(*n* = 67)	*P*-value
Age, years	64.61 ± 9.73	64.88 ± 9.35	64.39 ± 10.38	0.786
Gender, male	78 (65.5)	38 (73.1)	40 (59.7)	0.128
BMI (kg/m²)	25.10 ± 3.34	25.01 ± 3.20	25.17 ± 3.47	0.785
Hypertension	68 (57.1)	36 (69.2)	32 (47.8)	0.019
Hyperlipidemia	38 (31.9)	21 (40.4)	17 (25.4)	0.081
Smoking	26 (21.8)	17 (32.7)	9 (13.4)	0.012
Coronary heart disease	16 (13.4)	7 (13.5)	9 (13.4)	0.996
Diabetes	44 (37.0)	23 (44.2)	21(31.3)	0.149

The symptomatic group exhibited significantly greater stenosis severity, remodeling index, plaque burden, maximum plaque thickness, and eccentricity index than the asymptomatic group (all *p* < 0.001). The CCA-ICA angle and the proximal ICA curvature angle were significantly smaller in the symptomatic group than in the asymptomatic group (*p* < 0.001), whereas the bifurcation angle and the CCAmax/CCA ratio were significantly greater (*p* < 0.001). The prevalence of lipid-rich core plaques was significantly higher in the symptomatic group than in the asymptomatic group (*p* < 0.001). The maximum and mean PFD values on the plaque side were significantly higher in the symptomatic group than in the asymptomatic group, whereas no significant differences were observed in the corresponding contralateral PFD measurements between the two groups. Within both groups, the maximum and mean PFD values on the plaque side were significantly higher than those measured on the contralateral side (Table 2; Figure 3).

**Table 2:** Differences between symptomatic group and asymptomatic group.

Variables	Symptomatic(*n* = 52)	Asymptomatic(*n* = 67)	*P*-value	*χ*^2^/*Z* value
Plaque characteristics				
Plaque thickness (mm)	2.85 (2.48–3.62)	2.30 (1.90–2.65)	<0.001	−4.43^a^
Eccentricity index	0.67 ± 0.13	0.40 ± 0.18	<0.001	−7.18^a^
Degree of stenosis (%)	21.37 (15.95–30.33)	7.69 (4.70–14.05)	<0.001	−7.23^a^
Plaque burden (%)	72.50 ± 11.12	55.92 ± 11.35	<0.001	−6.57^a^
Remodeling index	1.60 (1.34–2.27)	1.19 (1.09–1.41)	<0.001	−5.35^a^
Geometric parameters				
Bifurcation angle	51.63 ± 4.95	42.41 ± 4.83	<0.001	−7.08^a^
CCA-ICA	141.75 ± 5.39	150.25 ± 5.34	<0.001	−6.65^a^
ICA	119.82 ± 9.04	133.47 ± 6.37	<0.001	−7.66^a^
CCA_max_/CCA	2.41 ± 0.30	1.99 ± 0.24	<0.001	−6.97^a^
Lipid-rich core plaques	47 (90.4)	11 (16.4)	<0.001	64.11^b^
Plaque side				
PFDmax(HU)	−37.95 ± 5.61	−49.74 ± 5.59	<0.001	−7.74^a^
PFDmean(HU)	−59.13 ± 5.12	−68.97 ± 5.12	<0.001	−7.46^a^
Opposite side				
PFDmax(HU)	−71.82 ± 3.54	−72.57 ± 3.87	0.601	−0.52^a^
PFDmean(HU)	−90.31 ± 4.46	−89.14 ± 16.18	0.151	−1.44^a^

a *Z* value.

b *χ* ^2.^

**Figure 3:**
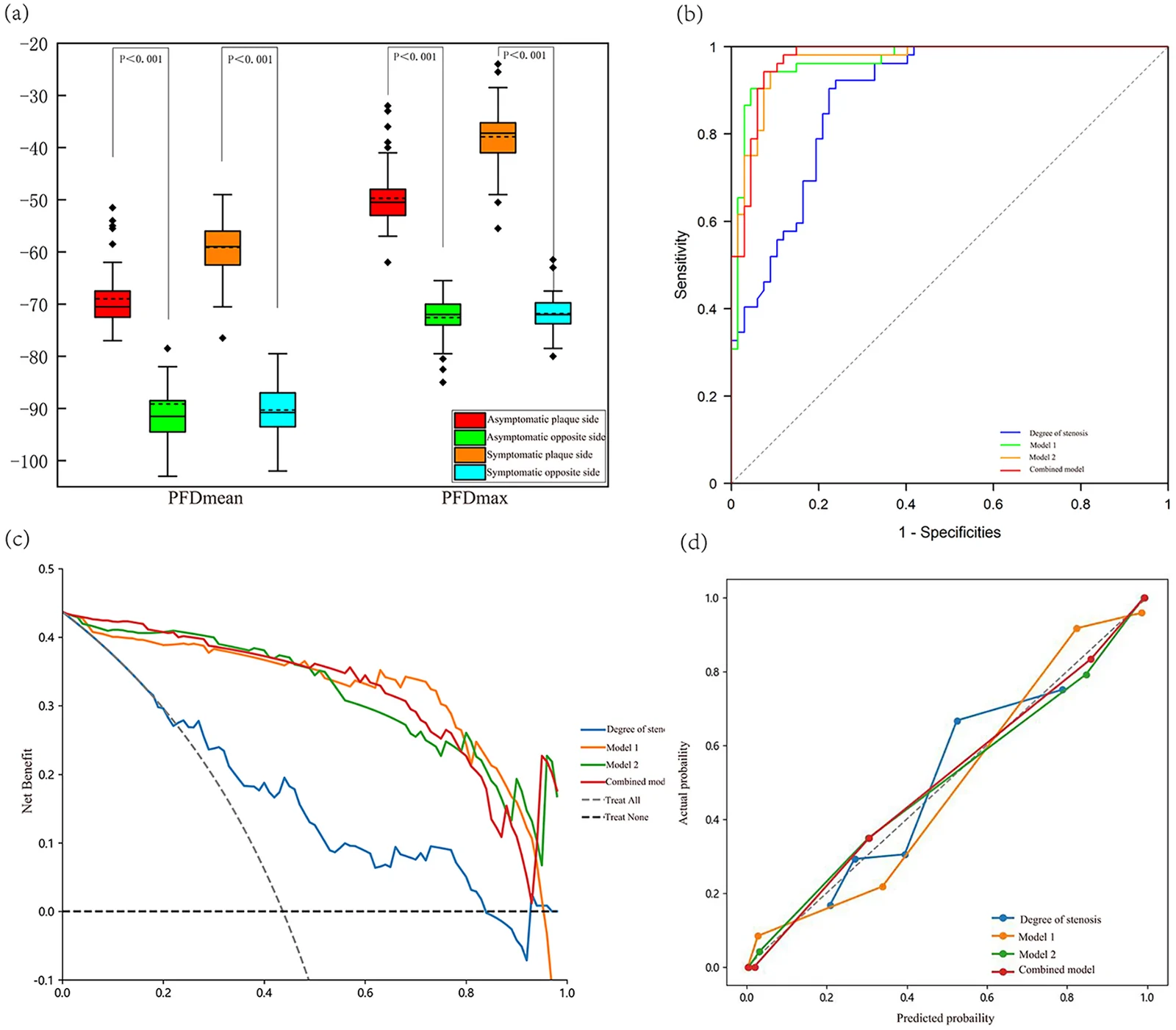
Comparison of perivascular fat density measurements and evaluation of model performance. Maximum and mean PFD values on the plaque side. **(b)** ROC curves for the discrimination of symptomatic carotid plaques. Model 1 included stenosis severity and LRNC; Model 2 included stenosis severity and maximum PFD; and the combined model included stenosis severity, LRNC and maximum PFD. **(c)** DCA of the discriminative models. The combined model provided the greatest overall net benefit across the evaluated threshold probabilities. **(d)** Calibration curves of the discriminative models. The solid red line represents the model-estimated probabilities, and the dashed line represents perfect calibration. Greater proximity of the solid line to the dashed line indicates better agreement between the model-estimated probabilities and observed event rates. Based on 119 patients (52 symptomatic vs. 67 asymptomatic).

Spearman rank correlation analysis demonstrated that stenosis severity, remodeling index, plaque burden, maximum plaque thickness, eccentricity index, and the presence of LRNC plaques were all positively correlated with both the maximum and mean PFD values (*p* < 0.001) (Table 3).

**Table 3:** Correlation between perivascular fat density and plaque characteristics.

Variables	*r* ^a^	*P*-value	*r* ^b^	*P*-value
Maximum plaque thickness	0.380	<0.001	0.436	<0.001
Lipid-rich core plaques	0.729	<0.001	0.782	<0.001
Eccentricity index	0.706	<0.001	0.747	<0.001
Remodeling index	0.432	<0.001	0.421	<0.001
Degree of stenosis	0.451	<0.001	0.442	<0.001
Plaque burden	0.633	<0.001	0.668	<0.001

a The maximum value of perivascular fat density.

b The mean value of perivascular fat density.

In the univariable analysis, all non-baseline variables entered into the multivariable model showed significant intergroup differences (*p* < 0.05), whereas the excluded baseline variables did not (*p* > 0.05). Furthermore, univariable logistic regression revealed that plaque characteristics, PFD parameters, the presence of LRNC plaques, and geometric parameters were each significantly associated with cerebrovascular events (all *p* < 0.001). After adjustment for clinical variables, multivariable logistic regression analysis demonstrated that stenosis severity (odds ratio [OR] = 1.287, 95% confidence interval [CI]: 1.116–1.485), the presence of LRNC plaques (OR = 10.344, 95% CI: 1.472–72.679), and maximum PFD (OR = 1.196, 95% CI: 1.045–1.369) remained independently associated with cerebrovascular events (*p* < 0.05) (Table 4). After the relevant factors were determined, multicollinearity was further assessed. The absolute values of pairwise correlation coefficients among all continuous variables were below 0.90, and the variance inflation factors (VIFs) for all factors in the multivariable logistic regression model were <5. These findings indicated no significant multicollinearity; consequently, all three factors were retained for subsequent model development and validation.

**Table 4:** Single-factor and multi-factor analysis.

Variables	Univariate logistic regression			Multivariate logistic regression		
	OR	95%CI	*P*-value	OR	95%CI	*P*-value
Plaque thickness (mm)	3.183	1.752-5.780	<0.001			
Eccentricity index	1.111	1.072–1.151	<0.001			
Remodeling index (%)	1.267	1.133–1.417	<0.001			
Degree of stenosis (%)	1.251	1.153–1.358	<0.001	1.287	1.116–1.485	0.001
Plaque burden	1.127	1.080–1.177	<0.001			
LRNC(mm^2^)	47.855	15.521–147.548	<0.001	10.344	1.472–72.679	0.019
Bifurcation angle	1.365	1.238–1.506	<0.001			
CCA-ICA(°)	0.764	0.697–0.836	<0.001			
ICA(°)	0.758	0.698–0.823	<0.001			
CCA_max_/CCA	1.734	1.441–2.086	<0.001			
PFDmax(HU)	1.369	1.238–1.515	<0.001	1.196	1.045–1.369	0.009
PFDmean(HU)	1.352	1.226–1.491	<0.001			

Discriminative models were constructed based on the independent correlates identified by multivariable logistic regression analysis, and their performance is summarized in Table 5. After internal validation, the AUCs for stenosis severity, LRNC plaques, and maximum PFD alone were 0.885, 0.863, and 0.908, respectively. Adding LRNC plaques or maximum PFD to the stenosis-only model (Models 1 and 2, respectively) increased the AUC to 0.966 and 0.971. The combined model, incorporating all three discriminators (stenosis severity, LRNC plaques, and maximum PFD), achieved the highest AUC of 0.972. At the optimal cutoff value of 0.571, the combined model yielded a sensitivity of 94.2% and a specificity of 92.5%.

**Table 5:** Assessment ability of all models.

Variables	Cutoff	AUC	optimism-corrected AUC(95%CI)	calibration slope	intercept	SE(%)	SP(%)	*P*-value
Degree of stenosis	14.07	0.887	0.885 (0.826–0.939)	0.9418	−0.0538	92.3	76.1	<0.001
LRNC	0.50	0.870	0.863 (0.807–0.928)	0.9327	0.0146	90.4	83.6	<0.001
PFDmax	−44.75	0.914	0.908 (0.846–0.956)	0.9674	0.0327	94.2	88.1	<0.001
Model 1	0.631	0.967	0.966 (0.932–0.993)	0.8614	0.0573	90.4	95.5	<0.001
Model 2	0.261	0.968	0.971 (0.938–0.992)	0.9570	0.0135	98.1	88.1	<0.001
Combined model	0.571	0.973	0.972 (0.944–0.995)	0.9186	−0.0277	94.2	92.5	<0.001

Model 1, degree of stenosis + LRNC; Model 2, degree of stenosis + PFDmax; Combined model, degree of stenosis + LRNC + PFDmax.

Pairwise comparisons of the AUCs among the four models were performed using DeLong's method (Table 6). Bootstrap analysis with 1,000 resamples (Figure 4) confirmed that Models 1, 2, and the combined model each significantly outperformed the stenosis-only model (all *P* < 0.05), whereas no significant differences were observed among Models 1, 2, and the combined model.

**Table 6:** Incremental value of the models for assessment.

Model comparison	*Δ*AUC	*P*-value	*Z* value
Model 1—Degree of stenosis	0.079	0.002	3.059
Model 2—Degree of stenosis	0.081	0.003	2.952
Combined model—Degree of stenosis	0.085	0.002	3.147
Model 2—Model 1	0.002	0.907	0.117
Combined model—Model 1	0.006	0.520	0.643
Combined model—Model 2	0.004	0.565	0.576

Model 1, degree of stenosis + LRNC; Model 2, degree of stenosis + PFDmax; Combined model, degree of stenosis + LRNC + PFDmax.

**Figure 4:**
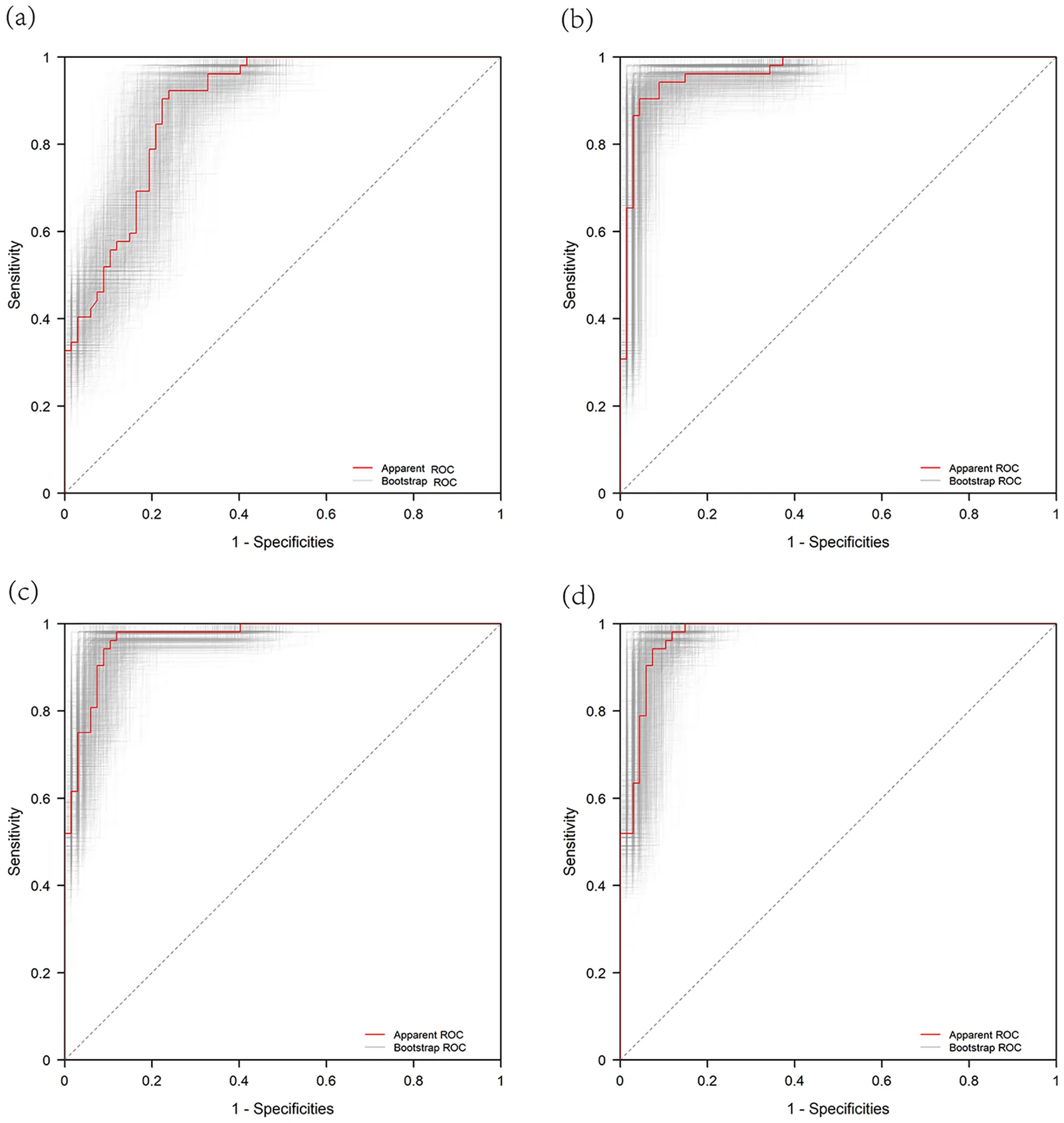
Bootstrap ROC curves (1,000 resamples) showing higher mean AUC for the combined model than degree of stenosis. **(a)** Degree of stenosis Bootstrap ROC curves (1,000 resamples). **(b)** Model 1 Bootstrap ROC curves (1,000 resamples). **(c)** Model 2 Bootstrap ROC curves (1,000 resamples) **(d)** Combined model Bootstrap ROC curves (1,000 resamples).

Calibration analysis demonstrated good agreement between the model-estimated probabilities and the observed event rates for Model 2 and the combined model, indicating better calibration than the stenosis-only model and Model 1. Decision curve analysis (DCA) further demonstrated that Model 1, Model 2, and the combined model provided greater net benefit than the stenosis-only model across the evaluated threshold probabilities (Figure 3).

## Discussion

Stroke remains a leading cause of mortality worldwide. Although CTA is widely used as a first-line clinical imaging modality for carotid artery assessment, its ability to characterize vulnerable non-calcified plaque components is limited by spatial resolution, partial-volume effects, and overlapping attenuation values. In addition, conventional CTA does not directly characterize local vascular inflammation. BBCT uses dedicated AI-assisted flexible registration and differential subtraction to suppress high-attenuation intraluminal contrast enhancement, thereby enabling black-blood visualization of the vessel wall. Delayed enhancement within the vessel wall and plaque tissue further enhances vessel wall-to-lumen contrast and facilitates the identification of LRNC plaques. PFD, an emerging imaging biomarker, may provide complementary information regarding local vascular inflammatory activity. In the present study, PFD was positively associated with carotid plaque characteristics. Furthermore, the model incorporating PFD, LRNC plaques, and stenosis severity demonstrated superior discriminative performance in identifying patients with recent symptomatic cerebrovascular events compared with the stenosis-only model, with good discrimination, calibration, and net clinical benefit.

Significant differences in plaque characteristics were observed between the symptomatic and asymptomatic groups. These differences may be attributable to distinct hemodynamic and inflammatory environments. Inflammation plays a central role in the initiation of low-density lipoprotein within the arterial wall promote endothelial activation, increased expression of adhesion molecules, macrophage-mediated lipid uptake, and foam cell formation. The resulting inflammatory cascade promotes plaque formation, arterial wall remodeling, and plaque instability. Inflammatory cytokines and proteolytic enzymes may also impair fibrous cap integrity, increasing plaque burden and the likelihood of plaque disruption (37). Progressive stenosis may further promote neovascularization, vessel wall hypoxia, and inflammatory responses, establishing a self-reinforcing process that may partly explain the differences in plaque characteristics between the two groups (38, 39).

Analysis of vascular geometry showed that the symptomatic group had a smaller CCA–ICA angle and proximal ICA curvature angles, but larger bifurcation angles and CCAmax/CCA ratios. One possible explanation is that a smoother vascular geometry reduces local wall shear stress and flow disturbances, thereby maintaining a relatively stable hemodynamic environment. Conversely, abnormal vascular geometry, particularly an increased bifurcation angle and a reduced curvature angle, may directly result in local hemodynamic disturbances, heterogeneous blood flow velocity distribution, and abnormal wall shear stress. These changes promote endothelial dysfunction, lipid deposition, and vascular inflammation, thereby accelerating the development and progression of carotid atherosclerosis (40).

The prevalence of LRNC plaques was significantly higher in the symptomatic group than in the asymptomatic group. LRNCs contain abundant free cholesterol, lipid material, and necrotic debris and are mechanically less stable than fibrous or calcified plaque components. An enlarged LRNC may increase mechanical stress on the fibrous cap and reduce the ability of the plaque to withstand cyclic hemodynamic loading (41). Inflammatory activity within and adjacent to the LRNC may impair collagen integrity and promote thinning or rupture of the fibrous cap (42, 43). These structural and biological characteristics may account for the observed association between LRNC plaques and cerebrovascular events.

Both the maximum and mean PFD values on the plaque side were higher than those on the contralateral side, with a more pronounced increase observed in the symptomatic group. This finding may reflect the interaction between vascular inflammation and adjacent PVAT. Inflammatory mediators released from the vessel wall may extend into the surrounding PVAT through the local tissue microenvironment. This process promotes adipose tissue lipolysis while inhibiting adipogenesis and adipocyte differentiation, resulting in increased CT attenuation of perivascular fat (44). Accordingly, higher plaque-side PFD values may indicate more pronounced local inflammatory activity, particularly in patients with recent ipsilateral ischemic cerebrovascular events.

Correlation analysis demonstrated that PFD was associated with multiple plaque characteristics. Because vascular inflammation is closely related to plaque characteristics, PFD may reflect local inflammatory changes associated with adverse plaque morphology. Bidirectional signaling between the vessel wall and adjacent PVAT may contribute to spatial concordance between intraplaque inflammation and perivascular fat attenuation. PFD can be measured using data acquired during routine carotid CTA without additional imaging sequences, radiation exposure, or contrast administration. PFD may therefore represent an accessible imaging marker for the assessment of local vascular inflammation and plaque-related risk.

Multivariable logistic regression analysis identified stenosis severity, the presence of LRNC plaques, and maximum PFD as independent correlates of cerebrovascular events. The various morphological plaque features do not exist in isolation but instead represent different manifestations of atherosclerotic progression and vascular remodeling. Their assessment may also be affected by measurement variability and plaque heterogeneity, making clinical interpretation more complex. In contrast, stenosis severity reflects the cumulative effects of multiple plaque characteristics and directly contributes to reduced wall shear stress, diminished perfusion, and decreased intraluminal pressure, thereby promoting hypoperfusion and plaque instability and providing an intuitive indicator of cerebrovascular event risk (45). In the present study, stenosis severity remained an independent correlates of cerebrovascular events after adjustment for clinical variables.

The presence of LRNC was also independently associated with cerebrovascular events. Owing to its low mechanical strength, an LRNC-containing plaque may be particularly susceptible to disruption under cyclic hemodynamic shear stress (46). Lipid and necrotic material may promote macrophage recruitment and activation, followed by the release of matrix metalloproteinases, including MMP-2 and MMP-9. These enzymes degrade collagen within the fibrous cap, thereby increasing the risk of plaque rupture (43). The combination of mechanical weakness and sustained inflammatory activity provides a plausible pathophysiological basis for association between LRNC plaques and cerebrovascular events. Since inflammatory responses are not uniformly distributed around the vessel wall but instead exhibit marked spatial heterogeneity, the most unstable and inflammation-active regions of the plaque are likely to induce more intense inflammatory responses in the adjacent PVAT, resulting in localized inflammatory hotspots.

Consequently, maximum PFD may more directly reflect the area of greatest local inflammatory activity than mean PFD (46). This finding further supports maximum PFD as an independent correlate of cerebrovascular events and suggests its potential utility for imaging-based risk stratification through the identification of localized inflammatory hotspots. However, whether changes in PFD contribute directly to the occurrence of cerebrovascular events remains unclear. Further studies are required to clarify this causal relationship.

Although the conventional model based solely on stenosis severity demonstrated limited discriminative ability for cerebrovascular events, its overall diagnostic performance remained suboptimal, limiting its utility for the precise identification of patients at high risk. In contrast, incorporation of LRNC into the stenosis model improved the overall performance of Model 1, whereas incorporation of maximum PFD improved the performance of Model 2. These findings propose that PFD, stenosis severity, and the presence of lipid-rich core plaques provide complementary information for the assessment of cerebrovascular events. The combined assessment captures the pathophysiological characteristics of carotid artery disease from multiple dimensions, thereby providing a more comprehensive and reliable basis for cerebrovascular risk stratification. The calibration curve for the stenosis-only model showed the greatest deviation from the reference line. This finding may reflect the inability of a single anatomical parameter to comprehensively characterize vessel wall pathology or fully represent the risk of cerebrovascular events. In contrast, the combined model integrated complementary data from multiple dimensions, thereby reducing the limitations associated with a single correlate and providing more accurate risk estimation across the full range of risk probabilities. DCA demonstrated that the combined model provided greater clinical net benefit than the stenosis-only model across the assessed threshold probabilities.

## Limitations

This study had several limitations. First, as a single-center retrospective cross-sectional study, it is subject to selection bias and cannot establish temporal precedence; reverse causation cannot be excluded, and findings should be interpreted as associations, not causal relationships. Generalizability requires validation in multicenter prospective cohorts. Second, manual delineation due to software limitations precluded volumetric quantification. Third, the relatively small sample size precluded stratified analyses of pre-CTA medication use, which may have introduced residual confounding. Fourth, the incremental discriminative value of imaging parameters was assessed against a stenosis-only model rather than a model including clinical risk factors; although clinical variables were balanced between groups, this may have overestimated the incremental contribution of imaging. DCA results were exploratory and lacked external validation. Although the empirical covariate screening strategy was supported by bootstrap validation and VIFs < 5, residual confounding may remain. Fifth, including only patients with unilateral carotid atherosclerosis may limit generalizability to bilateral disease. Finally, the lack of histopathological validation and prospective endpoints, together with strict inclusion/exclusion criteria, may further limit generalizability, warranting external validation.

## Conclusion

PFD was positively associated with carotid plaque characteristics and may serve as an imaging biomarker of local perivascular inflammatory activity. A discriminative model integrating maximum PFD, stenosis severity, and the presence of LRNC plaques demonstrated good performance in assessing the symtomatic status of recent cerebrovascular events, with improved risk stratification compared with assessment based on stenosis severity alone.

## Data Availability

The original contributions presented in the study are included in the article/Supplementary Material, further inquiries can be directed to the corresponding author/s.
